# Efficacy of low-dose zoster prophylaxis in patients undergoing allogeneic hematopoietic cell transplantation

**DOI:** 10.1038/s41409-019-0717-8

**Published:** 2019-10-15

**Authors:** Kristen Mascarenhas, Jennifer Berano Teh, Kelly Peng, Heeyoung Kim, Andrew Sy, Stephen J. Forman, F. Lennie Wong, Ryotaro Nakamura, Sanjeet S. Dadwal, Saro H. Armenian

**Affiliations:** 1 Department of Population Sciences, City of Hope, Duarte, CA USA; 2 Hyundai Cancer Institute, Children’s Hospital of Orange County, Orange, CA USA; 3 Department of Hematology and Hematopoietic Cell Transplantation, City of Hope, Duarte, CA USA; 4 Division of Infectious Diseases, Department of Medical Specialties, City of Hope, Duarte, CA USA

**Keywords:** Infectious diseases, Health services

## To the Editor:

Reactivation of varicella zoster virus (VZV) can occur in 20–53% of patients during the first year after allogeneic HCT, contributing to a high burden of infectious complications after HCT, and increasing utilization of medical resources and healthcare costs [1]. Accordingly, current guidelines recommend antiviral prophylaxis (e.g., acyclovir) for a minimum of 1 year after HCT, or until discontinuation of immunosuppression, whichever occurs later [1]. The recommended dose of acyclovir (800 mg twice daily [BID]) was derived from at least two randomized studies that demonstrated efficacy (≤5% reactivation) in the first year after HCT [1, 2]. However, this strategy is limited by a high risk of nephrotoxicity brought on by the concurrent use of antimicrobials or immunosuppressive medications used to treat graft versus host disease (GVHD) [1]. Currently, there is variability in clinical practice across transplant centers, with studies suggesting short-term efficacy of lower dose acyclovir in solid organ transplant and select patients undergoing HCT [3, 4]. As such, VZV prophylaxis at our institution has been acyclovir 400 mg BID, with a duration of at least 1 year after HCT or 3 months after discontinuation of immunosuppressive medications, whichever occurs later. The long-term efficacy of this approach has not been well-described.

We conducted a retrospective cohort study of patients who underwent allogenic HCT as adults (≥18 years old) at City of Hope (COH) between January 1, 2010 and December 31, 2015. COH and non-COH medical records were the primary source of information. We abstracted demographic data (age at HCT, sex, race/ethnicity), CMV serostatus, diagnosis (acute myeloid leukemia [AML], acute lymphoid leukemia [ALL], myelodysplastic syndrome [MDS], other), HCT comorbidity-age index (HCT-CI), Karnofsky performance score (KPS), HCT details (donor source, conditioning intensity [5]), relapse risk at HCT [6], severity of acute GVHD, and vital status per an established protocol [7, 8]. GVHD prophylaxis was tacrolimus/sirolimus-based or tacrolimus with a short course of methotrexate. Grading of acute GVHD was per established criteria [9]. The Institutional Review Board at COH approved the protocol. Informed consent was provided according to the Declaration of Helsinki.

The primary endpoint was VZV infection, categorized as dermatomal (involvement of 1–2 dermatomes) or disseminated (involvement of >2 dermatomes or extra-cutaneous involvement) per established definitions [10]. Patients were followed until the first episode of VZV infection or a competing risk event, whichever occurred first.

Univariate analyses were performed to compare between patients who developed VZV and those who did not. Relapse/progression or death was considered as a competing event. Time was calculated from the date of HCT to date of death, relapse/progression, or last contact (censored: December 31, 2018). Cumulative incidence of VZV infection was calculated taking into consideration competing risk for right-censored data [11]. Multivariable logistic regression analysis was performed to examine the modifiers of VZV infection risk (variables in the model: *p* < 0.2 in univariate analyses). Data were analyzed using SPSS 24.0 (IBM Corporation, Somers, NY). All statistical tests were two-sided and *p* < 0.05 was considered statistically significant.

There were 889 patients who met the eligibility criteria for the cohort. Median age at HCT was 52.3 (range, 18.0–78.6), and the most common indication for HCT was AML (40.8%), followed by ALL (20.5%), MDS (17.5%), and other (21.1%) diagnoses. The majority of patients were male (57.5%), at high risk for relapse at HCT (55.7%), and treated with reduced intensity conditioning (55.6%). Overall, patients had a low comorbidity burden (HCT-CI < 3, 75.6%), and a good functional status (KPS > 80, 65.4%) at HCT. HLA-matched (related or unrelated) stem cells were the donor source for 49.6% of patients, and 18.7% developed severe acute GVHD post-HCT. Median follow-up for the cohort was 3.2 years (range, 0–8.8 years), representing 2873 person-years of follow-up. Of the 424 (47.7%) patients who died during the follow-up period, all were followed until the VZV event, or death. Of the 465 (52.3%) patients known to be alive, 98% and 93% were followed for a minimum of 2 and 3 years, respectively.

**Fig. 1:**
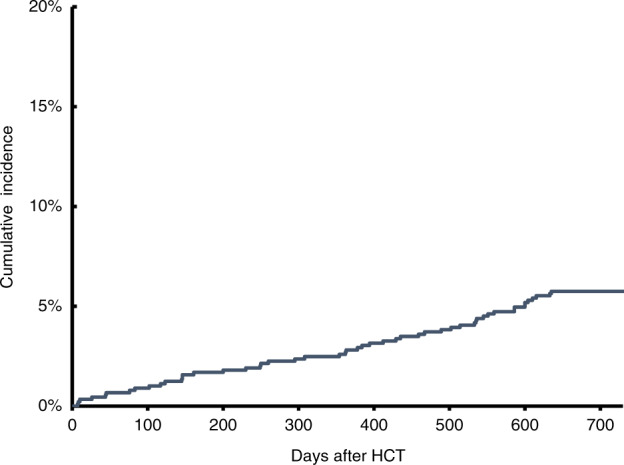
Cumulative incidence of varicella zoster virus reactivation after HCT

Fifty-one patients developed a VZV infection, at a median 1.03 years (range, 0.02–1.82) after HCT. Three patients had disseminated disease, and seven patients had reactivation <100 days from HCT. The cumulative incidence of VZV infection was 2.8% at 1 year, and 5.8% at 2 years following HCT (Fig. 1). Patients who developed VZV were significantly younger (44.3 years vs. 52.4 years; *p* = 0.015) at HCT, were more likely to have a diagnosis of ALL (35.3% vs. 19.6%, *p* = 0.045), and to be Hispanic (47.1% vs. 27.0%, *p* = 0.007), compared with those who did not develop VZV. None of these variables were statistically significantly associated with VZV reactivation in the multivariable regression model.

The principal finding from this study was that the use of lower dose acyclovir prophylaxis was associated with a low rate of VZV reactivation in the first year after HCT, with no evidence of clinically significant rebound at 2 years after HCT. Less than 1% of the cohort had reactivation <100 days from HCT, which represents the period of highest risk for reactivation after HCT. The rates reported in our study are comparable with, and in some cases lower, than those reported in studies that utilized higher dose acyclovir [2, 3]. These findings speak to the need to reconsider current VZV prophylaxis recommendations for this growing population of patients.

Previous studies have examined the efficacy of lower dose (total daily dose: <1600 mg) acyclovir prophylaxis in allogeneic HCT patients, with mixed results [3]. In these studies, the incidence of reactivation during the first year has ranged from 2 to 13%, with a high rebound rate ranging from 15 to 32% at 2 years after HCT [3]. While we are not able to speculate about the differences in outcomes between our study and others, it is possible that our requirement for continuation of low-dose prophylaxis up to 3 months after immunosuppressive medications may have contributed to the lack of rebound effect. A sizeable proportion of patients who had VZV reactivation in our study may not have been on optimal dosing as a result of concurrent infections that necessitated the use of other antivirals, renal insufficiency, or possible malabsorption due to gastrointestinal GVHD. Due to the shortcomings of prolonged oral prophylaxis, there have been increasing calls to consider scheduled vaccination starting 6 months after HCT [1]. The development of inactivated or recombinant zoster vaccine (RZV) has attenuated some of the concerns regarding live vaccines in immunocompromised patients [1]. While RZV has been effective in patients after autologous HCT [12], it has not been formally evaluated after allogeneic HCT. Studies are needed to examine whether a combination of lower dose acyclovir followed by RZV, given once off immunosuppressive medications, may be an effective strategy for allogeneic HCT survivors. Until then, our study suggests that the low-dose acyclovir prophylaxis approach employed at our center may effectively control VZV reactivation, without the associated late-occurring rebound seen with previous studies.
